# First Report and Genome Resource of *Monilinia vaccinii-corymbosi*, causal agent of Mummy Berry Disease of Black Huckleberry (*Vaccinium membranaceum*)

**DOI:** 10.7150/jgen.97432

**Published:** 2024-08-06

**Authors:** Rishi R. Burlakoti, Sanjib Sapkota, Mark Lubberts, Mehdi Sharifi

**Affiliations:** 1 Science and Technology Branch, Agassiz Research and Development Centre, Agriculture and Agri-Food Canada, Agassiz, BC, Canada.; 2 Science and Technology Branch, Summerland Research and Development Centre, Agriculture and Agri-Food Canada, Summerland, BC, Canada.

## Abstract

*Monilinia vacccinii-corymbosi* (phylum Ascomycota, family Sclerotiniaceae) causes fruit disease 'mummy berry' on berry crops and responsible for yield losses and quality of fruits. We reported mummy berry disease of black huckleberry (Vaccinium membranaceum) first time in British Columbia, Canada. We have performed sequencing and genome assembly of *M. vacccinii-corymbosi* from infected fruits of huckleberry. The resulting genome was 33.8 Mbp in size and consisted of 2,437 scaffolds with an N50 of 33,816 bp. To our best knowledge, this is the first report of resource announcement of whole genome sequence of mummy berry pathogen (*M. vacccinii-corymbosi*) infecting black huckleberry. The genome resource will be valuable for future studies to understand the genomic structure of pathogen, and mechanisms associated with black huckleberry-*M. vacccinii-corymbosi* interactions.

## Introduction

**Figure 1:**
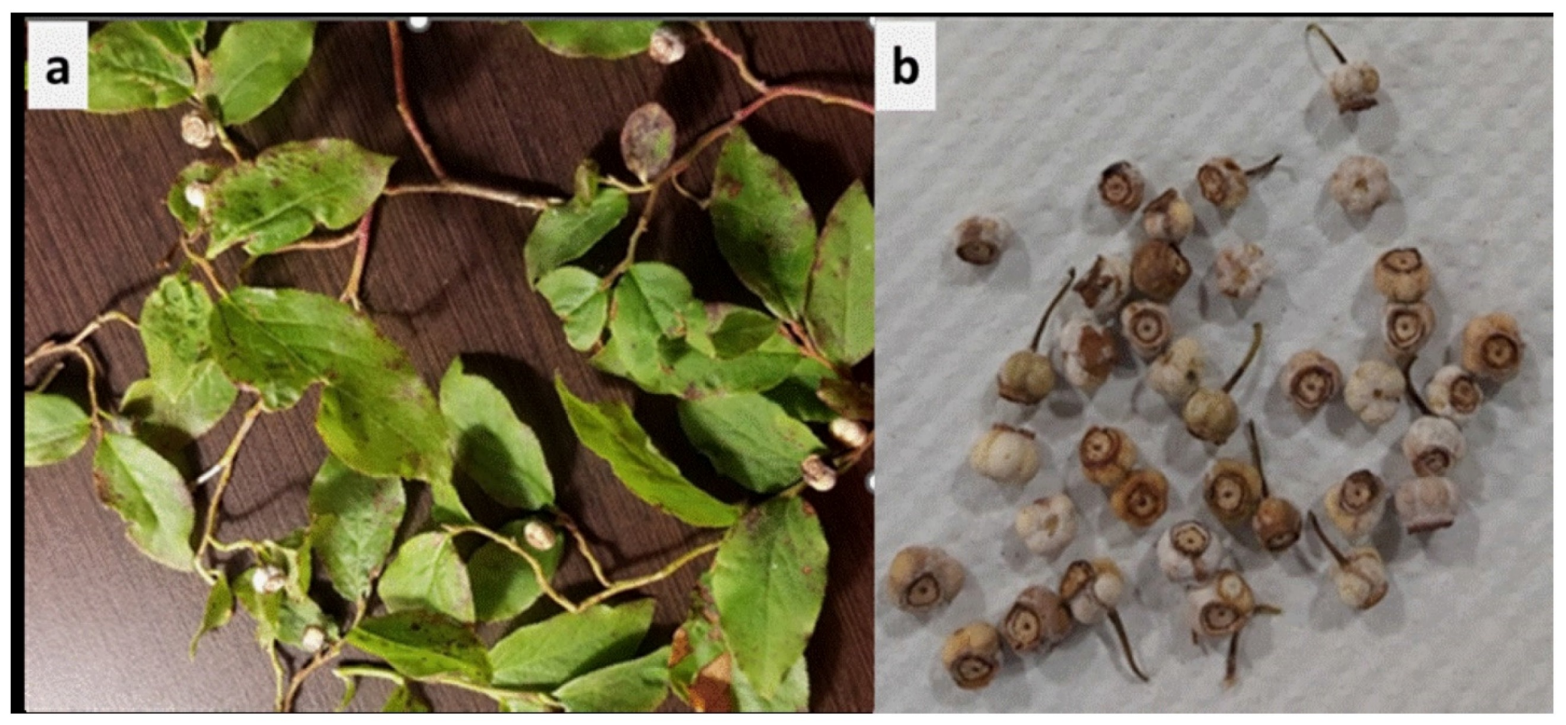
Symptoms of mummy berry of black huckleberry (*Vaccinium membranaceum*). **a)** Dried mummified berries on branches, **b)** Harvested dried mummified berries.

*Monilinia* spp. is a fungal genus belongs to the family Sclerotiniaceae of the phylum Ascomycota and several species of *Monilinia* cause economically important diseases on stone fruits, pome fruits and berry fruits 1,2. *Monilinia vacccinii-corymbosi* (Reade) Honey (hereafter referred as Mvc) causes mummy berry disease in both cultivated and wild species of blueberry 2. Mvc produces sexual spores, ascospores and asexual spores, conidia. The ascospores infect the young leaves in spring and produces abundant conidia in symptomatic foliage. These conidia infect berry flowers and fruits and develop mummified fruits, known as mummy berries in the main season, causing yield loss due to premature fruit drops and also make the fruit non-edible and unmarketable. *Monilinia* spp. was also reported to cause mummy berry disease on black huckleberry (*Vaccinium membranaceum*) in Oregon, USA 3. Black huckleberry is widely distributed and harvested wild berries in Pacific Northwest of USA and Canada, and the fruits are used as food for human and wildlife 4,5. In our study, we found dried mummified fruits in huckleberry plants (Fig. 1) from three sites (huckleberry patches) from Interior/Okanagan regions of British Columbia (BC). The causal agent of mummy berry of black huckleberry from BC were not identified previously. In addition, the genome of pathogen causing mummy berry of huckleberry were not studied elsewhere. This is the first resource announcement of whole genome sequence of mummy berry pathogen infecting huckleberry. The genome sequence will provide insight of genomic structure of the pathogen, biology and host-pathogen interaction.

## Materials and Methods

Genomic DNA was extracted directly from mummified berry samples of black huckleberry (Fig.1) using the MagMAX kit (ThermoFisher Scientific Inc.) and manufacturer's instructions was followed to extract genomic DNA. The genomic quality and quantity of DNA were measured using a Qubit 2.0 fluorimeter (Life Technologies Ltd., Paisley, UK). For sequencing, 2 x 150 bp paired-end configuration libraries were constructed using KAPA Hyperprep PCR-free library kit according to the manufacturer's instructions. Sequencing was performed on an Illumina HiSEQX next-generation sequencing device at Admera Health LLC (Plainfield, NJ) following the manufacturer's directions. A workflow utilizing the Nextflow management system 6 was devised for the assembly and annotation of whole genome. Initially, raw reads underwent quality and adapter trimming using BBduk. Subsequently, these reads were mapped to masked human and plant genomes using BBMap to eliminate host and contamination DNA. Following this, normalization and error correction were conducted utilizing BBNorm and Tadpole (https://jgi.doe.gov/data-and-tools/software-tools/bbtools/). The processed reads of Mvc were assembled using assembler Spades v3.15 7, and filtered for non-eukaryotic content using TIARA 8 (minimum length of 1000 bp) to identify and exclude potential non-eukaryotic co-isolates. The assembly was annotated using Funannotate v1.8.13 (https://github.com/nextgenusfs/funannotate) with the following associated software: Interproscan 9 and SignalP v6 10. Genome completeness was evaluated using BUSCO version 5.4.4 11, employing default parameters including the Eukaryota_Odb9 lineage dataset. Genes were predicted using BLAST (tblastn, 12) and Augustus (http://bioinf.uni-greifswald.de/augustus/). To enhance the accuracy of identifying conserved genes, any missing BUSCOs were reanalyzed using a comprehensive search approach.

## Results and Discussion

We found several shrivelled mummified fruits in many black huckleberry plants, typical symptoms of mummy berry on fruits in huckleberry patches in Okanagan (Fig. 1), indicating that mummy berry is a major fruit disease of black huckleberry. A metagenomic sequence classifier, Kraken2 version 2.0.8 13, was used to identify *Monlinia vacccinii-corymbosi* (Mvc) using a NCBI nucleotide database built on May 2nd, 2023 (https://benlangmead.github.io/aws-indexes/k2). The raw sequencing data were input into Kraken2, which rapidly analyzed and assigned taxonomic labels to the sequences based on the comprehensive nucleotide database. This approach accurately pinpointed the pathogen as *Monilinia vaccinii-corymbosi*, confirming the presence of the disease-causing agent. A total of 13.8 Gbp of Illumina reads were generated with an estimated 303x average depth of sequencing coverage. The assembled genome sequence was 33.8 Mbp in 2,437 scaffolds with an N50 of 33,816 bp. BUSCO completeness scores were 80.0% using the Letiomycetes gene set. The total number of predicted genes and proteins were 8,260 and 8,126, respectively. The genome size of Mvc from our study is similar to previously published genomes of Mvc strain isolated from blueberry in USA, 30.0 Mbp 14, while the genome size of *M. fructigena* (causal agent of brown rot) strain isolated from plum in southern Italy was slightly bigger, 43.1 Mbp 15.

Compared to Mvc strains from blueberry, a very little is known on the pathogen biology and genetic diversity of Mvc strains from the huckleberry. Hagerty *et al.* (2022) reported mummy berry in huckleberry in USA and the pathogen showed closet identity with Mvc strains based on sequence data of internal transcribed spacer (ITS) region, but they could not confirm the species and speculated that the pathogen could be new species of *Monilinia*. Using the whole genome sequence data, we confirmed Mvc as a causal agent of mummy berry huckleberry. This will be first genome resources of Mvc isolated from huckleberry and the genome data will be useful for further studies on genome annotation and comparison of Mvc strains from different hosts. In addition, the genome data will help in understanding the mechanisms and factors associated with interaction between Mvc and huckleberry plants.

## Figures and Tables

**Table 1:** Genome assembly statistics of *Monilinia vacccinii-corymbosi* causing mummy berry of black huckleberry (Vaccinium membranaceum) from our study compared with *M*. *vacccinii-corymbosi* isolated from southern highbush blueberry (V. darrowi) from Yow *et al.* 14.

Genome Assembly	*M*. *vacccinii-corymbosi* from our study	*M. vacccinii-corymbosi* from Yow *et al.* 2021
Assembled genome Size (Mbp)	33.8	30.0
Genome coverage	303 χ	413 χ
GC Content	40.73	44.53%
BUSCO Completeness	80.0%	98%
Numbers of predicted genes	8,260	9,399
